# Waiting times for diagnosis of attention-deficit hyperactivity disorder in children and adolescents referred to Italian ADHD centers must be reduced

**DOI:** 10.1186/s12913-019-4524-0

**Published:** 2019-09-18

**Authors:** Maurizio Bonati, Massimo Cartabia, Michele Zanetti, the Lombardy ADHD Group, Stefano Conte, Valeria Renzetti, Laura Salvoni, Massimo Molteni, Sara Trabattoni, Paola Effedri, Elisa Fazzi, Elena Filippini, Elisabetta Pedercini, Edda Zanetti, Nadia Fteita, Daniele Arisi, Roberta Mapelli, Simona Frassica, Simonetta Oriani, Christian Trevisan, Susanna Acquistapace, Ottaviano Martinelli, Davide Villani, Emanuela Binaghi, Andrea Deriu, Gabriella Vasile, Arianna Borchia, Paola Morosini, Maddalena Breviglieri, Giuseppe Capovilla, Roberto Segala, Chiara Battaini, Claudio Bissoli, Maria Paola Canevini, Isabella Cropanese, Emiddio Fornaro, Giorgio Leonardi, Silvia Merati, Laura Reale, Monica Saccani, Roberto Vaccari, Vera Valenti, Umberto Balottin, Matteo Chiappedi, Elena Vlacos, Corrado Meraviglia, Maria Grazia Palmieri, Gianpaolo Ruffoni, Francesco Rinaldi, Federica Soardi, Chiara Luoni, Giorgio Rossi

**Affiliations:** Laboratory for Mother Child Health, Department of Public Health, Istituto di Ricerche Farmacologiche Mario Negri IRCCS, Via Giuseppe La Masa 19, 20156 Milan, Italy

**Keywords:** Attention-deficit hyperactivity disorder, Children, Health service, Waiting time, Italy, Epidemiology

## Abstract

**Background:**

To investigate timely access to and the time needed to complete the diagnostic path of children and adolescents with suspected attention deficit hyperactivity disorder (ADHD) in the 18 Italian Lombardy Region ADHD reference centers.

**Methods:**

Data of children and adolescents enrolled in the Regional ADHD disease-oriented Registry for suspected ADHD who requested their first visit in 2013–2017 were analyzed.

**Results:**

The sample comprised 2262 children and adolescents aged 5–17 years who accessed the ADHD centers for diagnostic classification and management. The median waiting time was of 177 days (range 66–375) from the request for the initial appointment to the completion of the diagnostic path, with a three - fold difference between centers. In addition to the center, the strongest significant predictors of long waiting times were age comorbidities, the severity of the disorder, and having already completed some diagnostic procedures provided by the common standard path.

**Conclusions:**

To guarantee an equal standard of care in ADHD centers for all children and adolescents there is a pressing need to reduce the times to complete the diagnostic path. It is the task of both policymakers and each center to optimize the quality of the service and of the care delivered.

## Background

Accessibility, availability, and quality of care are important for efficient and effective healthcare systems [1, 2]. Prompt, and efficient service are the first requirements and expectations of patients who call on the National Health Service (NHS) [3]. Long waiting times are part of the structural barriers in all areas of the NHS, including mental health services, for children and adolescents, and for adults [4–6]. Patients suffering from mental health conditions who do not receive timely care, often experience a rapid decline in their conditions - with a lost opportunity for effective treatment [7, 8]. It is a challenge for policy makers and care providers to decide on the interventions needed to make the organization more effective, also reducing waiting times, although public mental health services are still not paying enough attention [9].

Long waiting times for a first appointment at Child and Adolescent Neuropsychiatric Services (CANPS) are one of the main reasons for user dissatisfaction with them [10]. A long wait can increase the patient discomfort - and distrust, and the intensity of symptoms, with a loss of motivation, non-attendance, and premature dropout [11]. The scarce resources for CANPS are one of the main reasons put forward to justify delays, and patients’ unfulfilled expectations [12]. Large differences in access and waiting times in CANPS have been reported in a few settings [13, 14] so they are to be expected in centers for attention-deficit hyperactivity disorder (ADHD) too.

ADHD is one of the complex neurodevelopmental disorders causing developmentally inappropriate and impairing patterns of inattention, hyperactivity, and impulsivity [15] that can have a dramatic impact on home and family life [16]. The reported range of prevalence is very wide (from 0.2 to 34.5%) with a worldwide estimate of 5.3% [17]. In Italy, when diagnostic care definition based on clinical evaluation is used as a definition, the estimated prevalence was 1.4% (from 1.1 to 3.1%) among the children aged 5 to 17 years [18]. Thus a considerable number of children and adolescents with suspected ADHD access CANPS for diagnosis.

In the Lombardy Region, where about 15% of the Italian pediatric population live, there is a network of 18 Regional ADHD reference centers, accredited by Regional health authorities as specialized ADHD hubs (Tier 3) of the CANPS network. A Regional ADHD Registry serves as a disease-oriented registry collecting information on all patients who access ADHD centers for diagnosis of suspected ADHD [19]. The Registry is part of a wider project aimed at ensuring appropriate ADHD management for every child and adolescent once the disorder is suspected and reported, and includes commonly acknowledged diagnostic and therapeutic procedures as well as educational initiatives for health care workers (child psychiatrists and psychologists) of the Lombardy Region health care system who provide assistance to ADHD patients and their families. Initiatives focused on increasing knowledge of ADHD in parents, teachers, and family pediatricians were also part of the regional project.

The main aim of this study was to examine waiting times for ADHD assessment in the 18 Regional ADHD centers, their variables, and inter-center differences for children and adolescents enrolled in the Registry between January 2013 and December 2017 for suspected ADHD.

## Methods

Data stored in ADHD Registry database were extracted and analyzed for the present study. Written informed consent was obtained for all patients before data collection. Data were anonymized prior to use for research. Formal ethical review board approval was not required for the present analysis. The study will be reported according to the published STROBE statement for prospective cohort studies (observational).

We used the methodology previously described and reporting details concerning: the local health setting [19], the characteristics of the ADHD Registry activated in Lombardy in June 2011 [20–22], the systematic work made by the18 ADHD centers belonging to the Lombardy ADHD Group [23], the rigorous diagnostic assessment (according to national and international guidelines, and *DSM*-*IV*-*TR*) approved by all involved clinicians [24, 25], the evaluation of follow-up and the effect size of provided care [26].

### Procedures

At the end of 2018 we searched the ADHD Registry to identify children residents in the Lombardy Region who requested their first visit to one of the 18 ADHD regional centers between 1 January 2013 and 31 December 2017, and completed the diagnostic procedure. We used spatial analysis to describe the distribution and ADHD center access according to place of residence, using the five-digit ZIP code to determine the local health protection agency (ATS) and the closest ADHD center for each child and adolescent. We assessed how the ADHD center characteristics and the anamnestic and clinical characteristics of the sample population contributed to the time needed for diagnosis, starting from the first request to the ADHD center.

### Statistical analyses

Geographic information system (GIS) software was used to generate maps (ArcGIS Desktop 10.3.1; Esri, Redlands, CA) that illustrate the geographic distribution of the homes of participants and the location of the ADHD center by centroid geospatial resource. The characteristics of the 18 ADHD centers were summarized using descriptive statistics.

Student’s t, Wilcoxon, and Kruskal-Wallis tests, and analysis of variance (ANOVA) were used to determine differences in population characteristics and between groups of subjects. Spearman’s rank correlation test was used to analyze the linear trend of wait times over the study period. Cross tabulations with chi-square, when appropriate, were done to explore the univariate associations.

A generalized linear model (GLM) analysis was also carried out to assess how the wait time affected completion as the diagnostic path (“estimated waiting time”). All tests were two-sided, and *p* < 0.05 was considered statistically significant. All analyses were done using SAS/STAT database (version 9.4, SAS Institute, Inc., Cary, NC, USA).

## Results

A total of 2464 children and adolescents’ residents in Lombardy Region required for the first time a visit for suspected ADHD during the period considered, 2262 (92%) had completed the diagnostic procedure at the time of data extraction, and were included in this study. These children and adolescents had a median age of 9 years at their first visit (range = 5–17 years; prevalence = 1.65 for 1000 residents of the same range of age), 1292 (85%) were males and 222 (15%) were females.

**Fig. 1:**
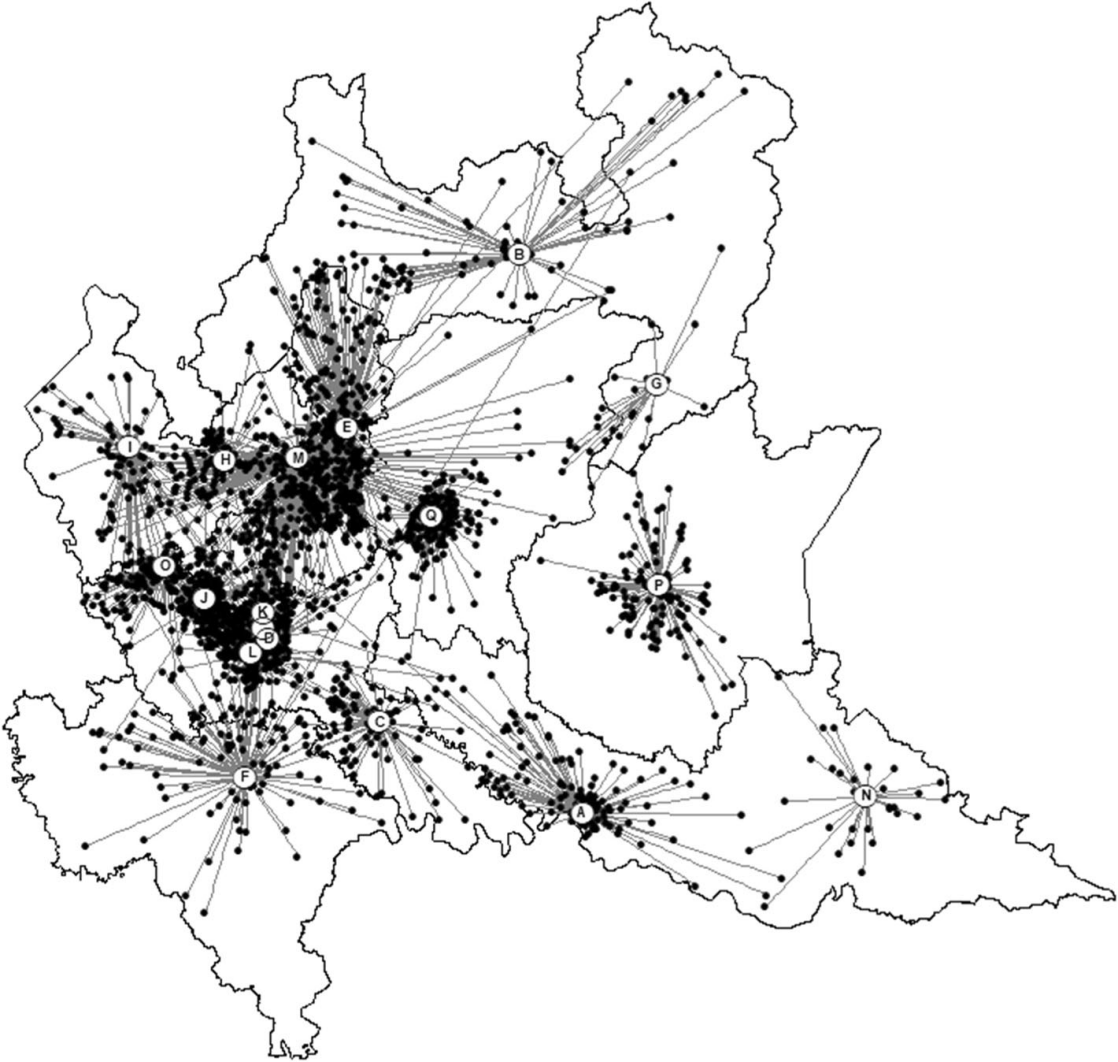
Euclidian distances between house of residence and accessed ADHD center. The map of Lombardy Region is divided according to the 8 ATS

The estimated rate of children requiring diagnosis varied across the 8 ATS with a range of 0.68–3.17 children for 1000 residents 5–17 years old. One thousand nine hundred seventy-nine children and adolescents (88%) attended an ADHD center within their ATS, 1726 (76%) the nearest their residence house, and 1656 (73%) attended the nearest ADHD center in their ATS of residence (Fig. 1). Of the 283 children and adolescents migrating across regional ATSs mean escape proportion was 5% (109/2262) ranging from 1 to 44% between ATSs, and a capture proportion of 7% (174/2262), between ATSs range 0–27%. The attraction index (capture/escape ratio) was highly positive (> 6.5) for two ATSs, and close 1 for one ATS, suggesting a varied capability of the Regional Health Service at ATS level answering residents’ requests of accessing ADHD centers.

The characteristics of the 18 referral ADHD centers for the Lombardy Region are described in Table 1. For all considered variables a wide variability between centers was found, not between years.

**Table 1:** Average characteristics of ADHD centers

Characteristics	mean (SD); median
No. of children and adolescents who accessed the Center per year	179 (90); 177
No. of children and adolescents who accessed the Center for the first time per year	35 (25); 32
No. of children and adolescents diagnosed with ADHD per year	24 (16); 20
No. of clinical staff professionals	5 (3); 5
Hours/year of work	1521 (620); 1473
Hours/year of work per children and adolescents with ≥1 access	11.5 (8.5); 8.3
Hours/year of work per children and adolescents with ≥1 access per clinical staff professionals	2.5 (1.9); 1.7
Wait time for the first visit (days)	112 (99); 82
Time from request to diagnosis (days)	204 (133); 177
No. of children and adolescents who accessed the Center per year	179 (90); 177
No. of children and adolescents who accessed the Center for the first time per year	35 (25); 32
No. of children and adolescents diagnosed with ADHD per year	24 (16); 20
No. of clinical staff professionals	5 (3); 5
Hours/year of work	1521 (620); 1473
Hours/year of work per accessed children and adolescents	11.5 (8.5); 8.3
Hours/year of work per accessed children and adolescents per clinical staff professionals	2.5 (1.9); 1.7
Wait time for the first visit (days)	112 (99); 82
Time from request to diagnosis (days)	204 (133); 177

**Fig. 2:**
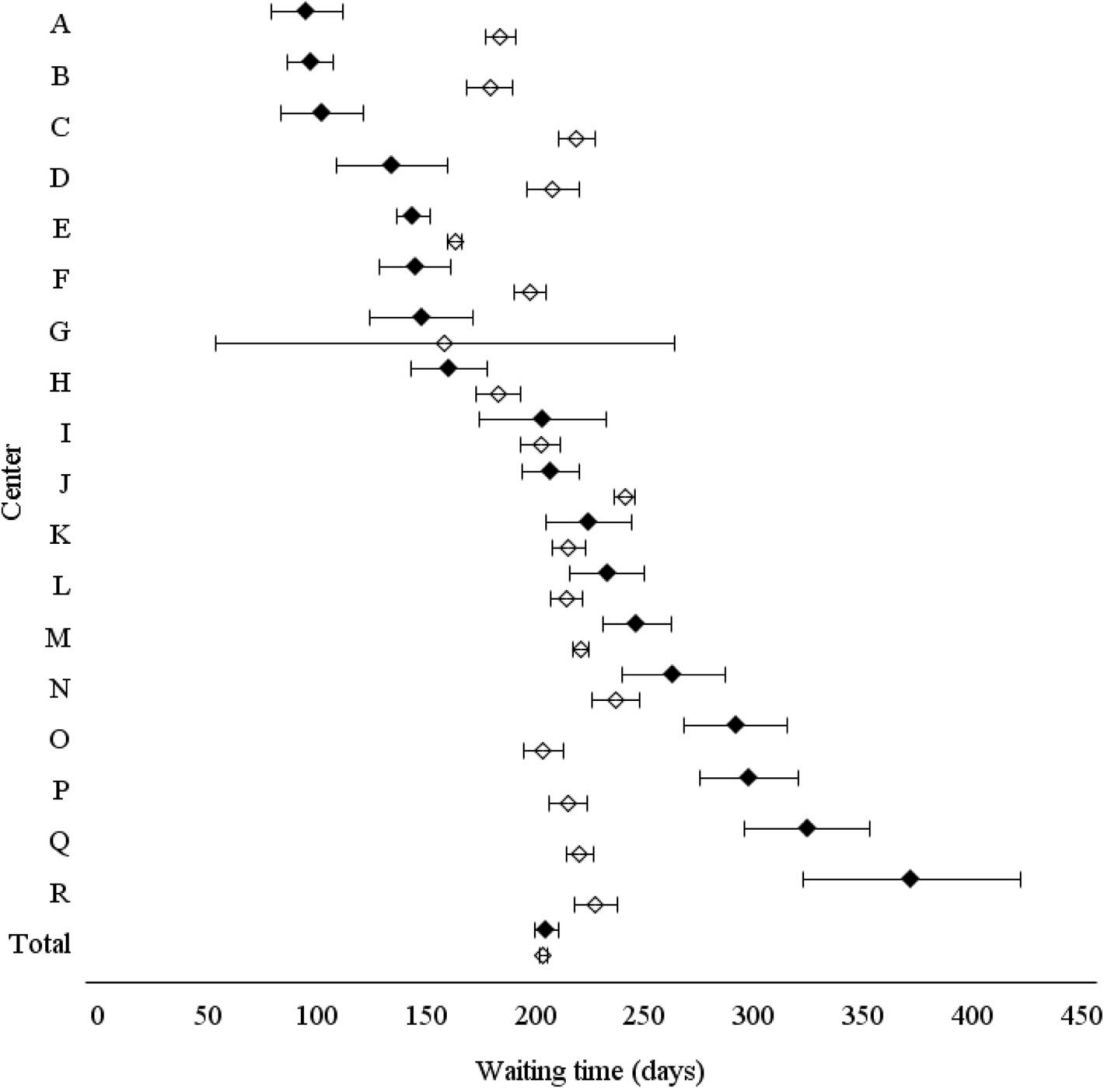
Time from request to diagnosis (days) by ADHD center. Observed (•) and estimated according to a generalized linear model (GLM) analysis (◊) values (mean, CI 95%)

**Fig. 3:**
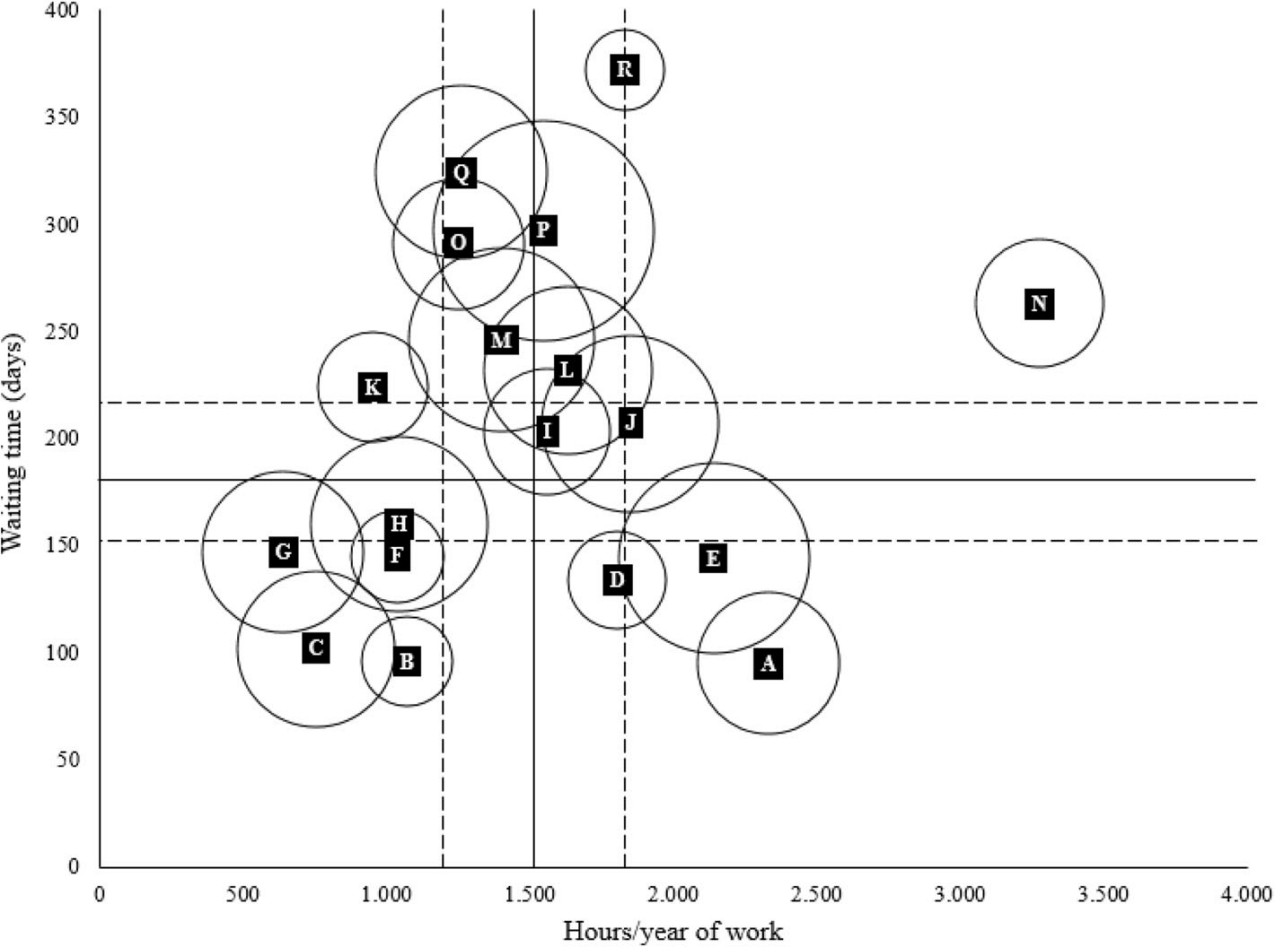
Distribution of ADHD centres by waiting time from the request to the diagnosis, the hours/year of work for ADHD, and the number of children and adolescents with ≥1 access per year. Mean (─), CI 95% (---). Footnotes: The size of circles is proportional to the number of children and adolescents per center included in the study. Pearson’s chi-squared test: ρ = 0.11; *p*-value = 0.6624; weighted Pearson’s chi-squared: ρ = − 0.10; p-value = 0.6789; Spearman’s rank correlation test: ρ = 0.01; *p* = 0.9838

Children and adolescents accessed the 18 ADHD centers for a median of 112 (range 25–396) youths per center. The range of median waiting time for the first visit was 14–212 days (overall 82), and of 66–375 days (overall 177) for the time from the request to the diagnosis without statistically significant differences between investigated years. A three times difference (F = 55.49, *p* < 0.0001) was observed between centers for the mean waiting time for completing the diagnostic trial (min 95 days, CI 95% 78–111; max 372 days, CI 95% 322–421; Fig. 2). No statistically significant relationship was observed between the waiting time from the request to the diagnosis, the amount of annual hours of work by clinical professional staff in each ADHD center, and the number of children and adolescents who accessed the center per year (Fig. 3). However, five centers (B, E, F, H, N) were outliers the 25th or 75th percentiles of all three variables, in particular B and F with all values lower than the 25th percentiles.

Of the 2262 children and adolescents in the study 1954 (86%) were diagnosed with a psychiatric disorder, and 1553 (69%) met *Diagnostic and Statistical Manual of Mental Disorders* (4th ed., text rev.; *DSM-IV-TR*; American Psychiatric Association [APA], 2000) criteria for ADHD diagnosis, whereas 151 (7%) had a chronic medical disease. As shown in Table 2, in addition to the difference between ADHD centers, the statistically significant characteristics of the population associated with a shorter waiting time for diagnosis by univariate analysis were: to have a younger age, a support teacher at school, a motor delay, another CANPS as referral, validated diagnostic tests already at the time of access to the center, a chronic medical disease, the access to the nearest ADHD center of the residence ATS, a middle-high CGI-S score at diagnosis. For attenders the centers with a psychiatric disorder average wait time was slightly longer than for children and adolescents without psychiatric disorders.

**Table 2:** Time from request to diagnosis (days) by characteristics of the sample population

Characteristics		N	Waiting time (days) [mean (SD); median]	*p*-value ^a^
Age at diagnosis	5–8 years	1088	194 (128); 163	0.0003
	9–17 years	1174	214 (137); 191	
Sex	Female	350	210 (136); 182	0.4110
	Male	1912	203 (132); 175	
Only child	No	1693	206 (132); 181	0.0925
	Yes	569	198 (135); 163	
Born abroad	No	2162	205 (133); 178	0.6219
	Yes	100	199 (132); 168	
Adopted	No	2190	205 (133); 178	0.2652
	Yes	71	188 (128); 157	
School repeater	No	2177	204 (133); 176	0.7265
	Yes	85	209 (138); 187	
Support teacher at school	No	2030	207 (132); 181	0.0009
	Yes	232	182 (135); 152	
Family history of ADHD	No	1873	203 (131); 173	0.2514
	Yes	389	213 (139); 193	
Born preterm or low weight	No	2029	205 (133); 178	0.2742
	Yes	233	195 (129); 167	
Motor delay	No	2146	206 (133); 180	0.0018
	Yes	116	172 (128); 130	
Language delay	No	1837	205 (132); 177	0.6040
	Yes	425	203 (136); 176	
Referral another CANPS	No	1539	229 (139); 213	< 0.0001
	Si	723	153 (100); 133	
Required assessment path done in the Center	No	1130	177 (128); 147	< 0.0001
	Yes	1132	232 (132); 210	
Optional evaluation done in the Center	No	788	208 (148); 182	0.5812
	Yes	1474	202 (124); 176	
Psychiatric disorders	No	308	187 (125); 159	0.0139
	Yes	1954	207 (134); 182	
Chronic medical diseases	No	2111	207 (134); 180	0.0010
	Yes	151	168 (110); 146	
Center in the ATS of residence and the nearest to the municipality of residence	No	625	191 (119); 163	0.0274
	Yes	1637	209 (137); 182	
CGI-S score at diagnosis	1–4	1810	208 (133); 181	0.0002
	5–7	389	183 (131); 155	

^a^: Wilcoxon or Kruskal-Wallis test

*SD* Standard Deviation, *ADHD* Attention-Deficit Hyperactivity Disorder. *CANPS* Child and Adolescent Neuropsychiatric Services, *ATS* Local health protection agencies, *CGI-S* Clinical Global Impression–Severity

Using linear regression analyses to determine response variables in population characteristics and centers, 6 of 10 statistically significant characteristics from univariate analysis were confirmed (the ADHD center, to be younger, to have been sent to the ADHD center by another CANPS, to have already done a few of mandatory diagnostic evaluations, to have chronic medical diseases, or to manifest severe symptoms of a disorder (CGI-S ≥ 5 at diagnosis), besides language delay associated with a longer waiting time from asking for the first visit to complete the diagnostic path (Table 3).

**Table 3:** Linear regression analysis for time from request to diagnosis (days) and characteristics of sample population

Parameter		Estimated waiting time (days) [mean (CI 95%)]	*p*-value ^a^
Intercept		18.4 (−38.5–75.2)	0.5264
Age at diagnosis	9–17 years vs 5–8 years	20.8 (10.0–31.5)	0.0001
Sex	Female vs Male	1.8 (−12.8–16.4)	0.8092
Only child	No vs Yes	5.2 (−7.0–17.5)	0.4022
Born abroad	No vs Yes	0.6 (−30.4–31.6)	0.9701
Adopted	No vs Yes	15.5 (−20.9–51.8)	0.4048
School repetear	No vs Yes	2.5 (−25.8–30.8)	0.8634
Support teacher at school	No vs Yes	4.3 (−14.3–22.9)	0.6508
Family history of ADHD	Yes vs No	3.3 (−11.1–17.8)	0.6507
Born preterm or low weight	No vs Yes	7.0 (−10.4–24.5)	0.4285
Motor delay	No vs Yes	16.5 (−8.2–41.1)	0.1899
Language delay	Yes vs No	15.8 (1.9–29.8)	0.0262
Referred by another CANPS	No vs Yes	64.6 (52.6–76.6)	< 0.0001
Required assessment path done in the Center	Yes vs No	43.8 (32.9–54.7)	< 0.0001
Optional evaluation done in the Center	No vs Yes	5.3 (−6.1–16.8)	0.3612
Psychiatric disorders	No vs Yes	8.8 (−7.0–24.7)	0.2750
Chronic medical diseases	No vs Yes	28.5 (7.5–49.6)	0.0079
Center in the ATS of residence and the nearest to the municipality of residence	Yes vs No	7.6 (−4.3–19.5)	0.2115
CGI-S score at diagnosis	1–4 vs 5–7	25.7 (11.0–40.4)	0.0006

^a^. Generalized linear model (GLM). r-squared = 0.12

*CI* Confidence interval, *ADHD* Attention-Deficit Hyperactivity Disorder, *CANPS* Child and Adolescent Neuropsychiatric Services, *ATS* Local health protection agencies, *CGI-S* Clinical Global Impression–Severity

Estimated waiting times values according to GLM analysis were more closed than observed (min 158 days, CI 95% 53–263; max 241 days, CI 95% 236–245; Fig. 2). No statistically significant difference between center observed vs estimated values was found for 5 centers (G, I, K, L, N), whereas for center A and B close and similar values were maintained between center.

## Discussion

To the best of our knowledge, this study, as part of a multimodal project, represents the first evaluation in a large population of the time needed to complete a diagnostic evaluation in different ADHD centers related to center and patient characteristics. To dwell on this variable (waiting time) of the care pathway is important because complex neurodevelopmental disorders (such as ADHD, autism spectrum disorders, tic disorders and Tourette’s syndrome, as well as learning disorders and intellectual impairment) can have a dramatic impact on home and family life and timely access to CANPS is expected by everyone.

The major findings of the present study can be summarized as follows: despite the common work shared over time a three times difference of waiting time for the similar diagnostic pathway was observed between centers. The size of waiting times between centers can be halved weighting for a few characteristics of children and adolescents attending each center. However, between center differences remain wide.

The results therefore indicate that a critical and shared comparison of the centers organization can be done to make waiting times more homogeneous between centers. Consequently, critical points should be tackled in the individual centers through effective initiatives for improving and maintaining over time the quality of care. On the other hands at ATS level interventions for improving the effectiveness of care delivery are needed. The local distribution of ADHD centers should be reviewed by the Regional Ministry of Health since 27% of population attended a different center than that provided by the organization of regional services network. This affects the distribution of workloads of the individual centers, causes family dissatisfaction with the Health Service, and increases the costs attributable to the greater distance from residence that the family has to cope with [4–6]. It is conceivable that it is not an exclusive situation for ADHD but for also the other disorders belonging of CANPS [7, 27, 28]. So what has been done and documented here for ADHD in specialized centers should be generalized to all CANPS, at least at the regional level, so that policy makers can intervene appropriately.

The complexity of health care systems can produce inefficiencies in healthcare delivery, in particular in health areas such as mental services where organization settings and people involved are often loosely connected. Various approaches, such as the sociotechnical system [29] or the quality assessment models [30], can be used to identify the elements cause of performance difference between centers, their interactions and their impact on quality care, as well as understanding the key adaptive role of people in the system [29]. However, sociotechnical system theory and interventions need more evaluation in child psychiatric area, as well as in Italy where a public universal health system has been up and running for decades in an efficient way. Different CANPS strategies and models of services delivery have been proposed or endorsed [11, 31–33], also for ADHD oriented services [16, 34–36], to facilitate access, reducing barriers, and improving facilitators. Signs of improvement have been seen but the rate of progress is still not good enough [14]. Waiting time reduction initiatives should be considered to answer the question “wait time to what?”, with the aim that shortened waiting time should improve the quality of delivered care, obtaining better clinical outcomes that must be monitored [37].

However, a mandatory intervention for each center is to manage better their available resources as suggested from not having found any association between the population size of attenders, the hours of work dedicated to ADHD diagnosis and therapeutic paths, and the waiting time.

A first and essential intervention to reduce differences between centers in the access should be the definition and utilization of common criteria of attitude and practice since the first request for an appointment. Appropriate prioritization, using different criteria (i.e. for ‘emotional disorders’ and for ‘developmental disorders) were set up and used in a real-world setting providing effective care of ADHD [16]. Different criteria for prioritization as well as for other psychiatric disorders are required and the findings of the present study give some indication. Children and adolescents referred by another CANPS or with part of the common regional diagnostic plan already performed elsewhere had to wait less than the other attenders, suggesting that it is important to maintain and implement the relationships and information exchanges between the CANPS, as well as between professionals working in the same territory of the ADHD center. The age, language delay, or chronic medical diseases can be part of the essential points of an appropriate triage (also by phone) to better plan the diagnostic path also in consideration of the availability and temporal possibilities of the Center.

The study setting is part of a larger, multimodal project, and represents a distinctive tool for ensuring appropriate and shared diagnostic and therapeutic pathways of care in ADHD children between the 18 participating centers. This homogeneity of the diagnostic and therapeutic practice between centers is a strength to evaluate other dependent variables, as waiting time, that can affect the care.

However, there is also the limitation that our findings refer specifically to the population accessing ADHD centers since only these hubs input data into the Registry about patients attending the center. Thus, although the 18 ADHD centers are public and cover the whole Region, it was not possible to evaluate initial appointment failures with patient losses.

It was not the aim of the study to evaluate if waiting time affected the outcome of the therapy then undertaken, although timely access to healthcare is associated with improved health outcomes [4, 38]. Besides, we know that early interventions in a period of development are crucial, and a prompt, preventive, and detection action is imperative for mental health disorders also in children and adolescents, and those with ADHD [39, 40].

## Conclusions

To guarantee an equal standard of care in ADHD centers for all children and adolescents the management of ADHD must stay within a pathway that strives for optimal care. The first step is the access to the ADHD center, thus long waiting times for diagnosis are a sign of poor care, or at least of a questionable care, since its inception. Besides, long waiting times have a negative impact on family satisfaction, staff moral, and referrer’s opinion of the service. Last but not secondary, differences in waiting times for the same needs (characteristics of children and adolescents attending the ADHD centers) are a sign of inequalities. A few critical points affecting waiting times have been highlighted in the present study. There is therefore reason and duty to intervene and improve the care.

## Data Availability

ADHD Registry database was created, developed, updated, and managed by the Authors within the project. The datasets used and/or analyzed during the current study are not public but available from the corresponding author on reasonable request for research pourposes.

## Abbreviations

ADHD: Attention-deficit hyperactivity disorder
ANOVA: Analysis of variance
ATS: Local health protection agency
CANPS: Child and Adolescent Neuropsychiatric Services
CI: Confidence interval
DSM-IV: Diagnostic and statistical manual of mental disorders
GIS: Geographic information system
GLM: Generalized linear model
SD: Standard deviation
